# Nuclear Magnetic Resonance–Based Urinary Metabolic Profiling for Assessing Heat Stress–Related Incident Kidney Injury in Health‐Care Workers

**DOI:** 10.1029/2025GH001583

**Published:** 2026-06-02

**Authors:** Cheng‐Yu Ting, Chi‐Tsung Chen, Tzu‐Hsin Yen, Jason Kai Wei Lee, Sheng‐Han Lee, Shang‐Jen Chang, Tzu‐Han Hung, Hao‐Jan Liang, Hsiao‐Yu Yang, Ching‐Yu Lin

**Affiliations:** ^1^ College of Public Health Institute of Environmental and Occupational Health Sciences National Taiwan University Taipei Taiwan; ^2^ Department of Respiratory Care Shin Kong Wu Ho Su Memorial Hospital Taipei City Taiwan; ^3^ Heat Resilience and Performance Centre Yong Loo Lin School of Medicine National University of Singapore Singapore Singapore; ^4^ Human Potential Translational Research Programme Yong Loo Lin School of Medicine National University of Singapore Singapore Singapore; ^5^ Department of Physiology Yong Loo Lin School of Medicine National University of Singapore Singapore Singapore; ^6^ School of Medicine College of Medicine National Sun Yat‐Sen University Kaohsiung Taiwan; ^7^ Department of Urology National Taiwan University Hospital Taipei Taiwan; ^8^ Population Health Research Center National Taiwan University Taipei Taiwan

**Keywords:** heat stress, incident kidney injury, nuclear magnetic resonance, metabolomics, trimethylamine N‐oxide, taurine

## Abstract

During the COVID‐19 pandemic, heat stress (HS) was a serious occupational risk for health‐care workers (HCWs) wearing personal protective equipment, which limits heat dissipation and increases the risk of kidney injury. In this exploratory study, we assessed HS‐related changes in the urinary metabolites of 38 HCWs working at mobile COVID‐19 screening units in Taiwan and their possible role in incident kidney injury (IKI). Urine samples were collected before and after their shifts. Nuclear magnetic resonance‐based metabolomics was applied to measure urinary metabolic perturbations associated with HS, and these findings were subsequently correlated with clinical markers of IKI. Data were analyzed through multivariate and univariate analyses. We found that HS‐related changes in taurine and trimethylamine N‐oxide (TMAO) levels showed a trend associated with postshift IKI. Preshift urinary citrate, acetone, and 4‐hydroxybenzoate levels tended to be higher in the IKI population than the non‐IKI population. Compared to the non‐IKI population, the IKI population exhibited a trend toward lower postshift levels of glutarate and valine, but higher postshift levels of citrate, taurine, TMAO, and histidine. Metabolites whose levels were altered after HS included creatine, methylguanidine, formate, methylmalonate, 4‐hydroxybenzoate, adenine, hippurate, and tryptophan. Our preliminary findings suggest that higher postshift urinary taurine levels in the IKI population are potentially related to oxidative stress. In contrast, elevated TMAO levels possibly indicate disturbances in the gut‐kidney axis affecting renal health. Higher baseline urinary citrate and acetone may suggest increased susceptibility to HS‐related IKI. Finally, elevated urinary glutarate in the non‐IKI population may represent a protective metabolic response.

## Plain Language Summary

During the COVID‐19 pandemic, health‐care workers faced intense heat stress (HS) from wearing heavy personal protective equipment (PPE) for long hours, increasing their risk of kidney injury. However, what happens inside the body during HS‐related kidney injury remains unclear. This study analyzed urine samples from health‐care workers in Taiwan, both before and after their shifts, to identify molecular “clues” that may be related to kidney injury. We found that workers with early signs of kidney injury had higher levels of two molecules, taurine and trimethylamine N‐oxide, after their shifts. This may be linked to the body's response to stress and an imbalance in gut bacteria. We also noted that individuals with higher levels of urinary citrate, acetone, and 4‐hydroxybenzoate before work seemed to be more vulnerable to heat‐related kidney injury. Our work describes potential metabolic changes associated with HS in health‐care workers. If confirmed in larger‐scale studies, these molecular clues could help us better understand who is most at risk. This study could also lead to the development of targeted preventive strategies, such as improving working conditions or providing nutritional support, to protect the health of these workers.

## Key Points

- Heat stress‐related metabolic changes observed in the postshift urine of health‐care workers
- Higher urinary taurine and trimethylamine N‐oxide in postshift kidney injured workers suggest protective and disruptive metabolic roles
- Higher baseline citrate, acetone, and 4‐hydroxybenzoate may suggest an increased risk of incident kidney injury

## 1 Introduction

Heat stress (HS), which occurs when the body struggles to manage heat from metabolism or the environment, poses substantial health risks (Epstein & Moran, 2006). It results from environmental heat and humidity (Zhang et al., 2023), increased physical activity (McGinn et al., 2017), and respirator and protective clothing use (Fang et al., 2023). Approximately 15% of all individuals exposed to high temperatures for a long duration develop kidney disease or acute kidney injury (AKI), characterized by a 14.5% increase in urine's specific gravity, dehydration, and early kidney stress (Flouris et al., 2018). Epidemiological evidence suggests that extreme heat considerably increases the risk of occupational injuries across climates; this risk increases by 1% for every 1°C rise in ambient temperature and by 17.4% during a heat wave (Fatima et al., 2021).

HS is associated with cardiovascular mortality (Cheng et al., 2019; Liu et al., 2022) and kidney diseases, including AKI (Flouris et al., 2018). The body mitigates heat through vasodilation and sweating. Physical activity, dehydration, and age increase HS and thus kidney injury risks (Butler‐Dawson et al., 2019; Chang & Yang, 2023; Chapman et al., 2021; López‐Gálvez et al., 2021). A US study demonstrated that higher plasma creatinine and osteopontin levels were associated with chronic heat exposure during 8.5‐hr shifts among agricultural workers, suggesting that HS increases the risk of AKI (Chicas et al., 2023).

Evidence from animal models suggests that the HS‐related increases in core body temperature is associated with mitochondrial dysfunction, reduced glomerular filtration rate, and disruptions to key metabolic pathways including gluconeogenesis and lipid metabolism (Sato et al., 2019; Xue et al., 2021; Yue et al., 2020). Furthermore, metabolomic analyses of heat‐stressed agricultural workers revealed impaired gluconeogenesis and histidine metabolism pathways, which may be associated with recurrent proximal tubule injury related to chronic HS (Chicas et al., 2023).

Metabolomics, a robust tool for analyzing small molecules, provides insights into the metabolic status of organisms (Nicholson & Lindon, 2008). Metabolomic approaches have been applied in various domains, such as basic biology, environmental toxicology, and clinical medicine (Huang et al., 2022). Our previous study involving health‐care workers (HCWs) working 2‐hr shifts during the COVID‐19 pandemic demonstrated increased HS from wearing personal protective equipment (PPE) or working outdoors (Chang et al., 2022). HS was significantly associated with reduced estimated glomerular filtration rate (eGFR) (*P* < 0.001), increased blood creatinine levels (*P* < 0.001), and elevated proteinuria risks, suggesting an increased risk of kidney injury (Chang et al., 2022). However, the potential metabolic perturbations associated with HS‐related kidney injury remain unclear. In the present study, we explored the urinary metabolic profiles of heat‐stressed HCWs and investigated potential metabolic biomarkers associated with HS‐related incident kidney injury (IKI). Data were collected from HCWs who worked wearing PPE during the COVID‐19 pandemic.

## 2 Materials and Methods

### 2.1 Study Cohort and HS

In our previous study (Chang et al., 2022), we recruited (between September and October 2021) 39 HCWs from mobile COVID‐19 screening units in Northern Taiwan. Past medical history was collected via a self‐administered questionnaire and physician interview. Because participants were health‐care professionals, information bias in self‐reported history was expected to be lower. The self‐controlled (pre‐ vs. postshift) design allowed each participant to serve as their own control. This within‐subject design inherently reduces confounding from stable individual characteristics (e.g., sex, baseline health status, and chronic conditions such as diabetes or hypertension) that remain constant during the study period. Participants were not excluded based on pre‐existing conditions or medication use to enhance the generalizability of the findings to the broader HCW population. The final cohort included 38 HCWs because the pre‐ and postshift urine samples of one nurse were insufficient for analysis. We used data from this cohort in the present study. We adopted a self‐controlled, repeated‐measures design to minimize interpersonal variability and enhance sensitivity for detecting HS‐related metabolic changes. Preshift measurements served as a baseline control. Data on the HCWs' demographic characteristics, work shifts, and physical characteristics were obtained from our previous study (Chang et al., 2022). Temperature and humidity inside the PPE were monitored using a Kestrel DROP D3 Logger (Nielsen‐Kellerman, Boothwyn, PA, USA), and ambient and personal heat indices were calculated for each shift. The heat index (HI) for both ambient and personal conditions was determined using the multiple regression equation developed by Lans P. Rothfusz (1990) and utilized by the U.S. National Weather Service. The formula is as follows:

$$\text{HI}=-42.379+2.04901523\times T+10.14333127\times \text{RH}-0.22475541\times T\times \text{RH}-0.00683783\times T^{2}-0.05481717\times {\text{RH}}^{2}+0.00122874\times T^{2}\times \text{RH}+0.00085282\times T\times {\text{RH}}^{2}-0.00000199\times T^{2}\times {\text{RH}}^{2}.$$

where *T* represents dry‐bulb temperature in degrees Fahrenheit (°F) and RH is the relative humidity (%). A specific adjustment is added to the HI for conditions where *T* is between 80 and 87°F and RH exceeds 85%. Furthermore, to account for solar radiation, a 7.5°C correction was added to the personal HI for participants working in direct sunlight, in line with OSHA recommendations (OSHA, 2022). During the study period, the average environmental temperature was 29.3°C ± 2.3°C. The ambient HI was 35.0°C ± 3.8°C. The personal HI, measured when the HCWs were using PPE at work, ranged from 46.8°C to 48.4°C (Chang et al., 2022).

To evaluate kidney function, whole blood creatinine levels were measured using fingertip blood samples collected before and after work. IKI was defined based on the Kidney Disease: Improving Global Outcomes Acute Kidney Injury Work Group, 2012 guidelines for AKI, namely, by a >0.3‐mg/dL increase or a >1.5‐fold increase in whole blood creatinine level relative to baseline (Kidney Disease: Improving Global Outcomes Acute Kidney Injury Work Group, 2012). To overcome field‐setting challenges, we employed a Point‐of‐Care Testing approach using the StatSensor® creatinine analyzer (Nova Biomedical, Waltham, MA, USA), which is widely used in emergency rooms and intensive care units in major hospitals throughout Taiwan. This device is well‐validated for such applications, demonstrating high concordance (88%) with traditional laboratory methods (Lemoine et al., 2022), excellent sensitivity (100%) with moderate specificity (79%) for community screening (Minnings et al., 2015), and verified clinical reliability with a concordance coefficient of 0.97 (Kosack et al., 2015). The study was approved by the Research Ethics Committee of National Taiwan University (No. 202106HN031). Research participants consented to the publication of results from this study.

### 2.2 Sample Collection and Preparation

A total of 75 urine samples were collected from 38 HCWs both before and after work. The samples were stored in labeled tubes containing sodium azide (final concentration: 0.05% wt/vol) to prevent bacterial contamination, immediately cooled on dry ice, and stored at −80°C until analysis (Beckonert et al., 2007).

For nuclear magnetic resonance (NMR) spectroscopy, 400 μL of thawed urine was mixed with 200 μL of sodium phosphate buffer (pH 7.4) in heavy water, containing 0.5 mM trimethylsilyl propionic acid (TMSP) and 3 mM sodium azide for pH stabilization (Beckonert et al., 2007). The mixture was vortexed for 20 s and then centrifuged at 12,000 × *g* for 5 min at 4°C. Then, 550 μL of the supernatant was transferred to a 5‐mm NMR spectroscopy tube for analysis. A quality control sample was prepared to ensure experimental consistency (Beckonert et al., 2007). The sample was prepared by pooling 10‐μL aliquots from each urine sample to create a composite that represented the average metabolome of the cohort.

### 2.3 ^1^H NMR Spectral Acquisition and Processing

The ^1^H NMR experiment was performed using a Bruker Neo 600 MHz NMR Spectrometer with a TXI cryoprobe (Bruker BioSpin, Ettlingen, Germany) at 300 K at the High‐Field NMR Center, Academia Sinica, Taiwan. A nuclear overhauser enhancement spectroscopy pulse sequence (d1‐90°‐t1‐90°‐tm‐90°) was used to suppress strong water resonance (Beckonert et al., 2007). Each spectrum was recorded with 32,768 data points across a spectral width of 12,000 Hz over 128 scans; the acquisition time was 1.63 s. An exponential line‐broadening factor of 0.3 Hz was applied before Fourier transformation to improve spectral quality (Beckonert et al., 2007). The width of the TMSP resonance line at half height was maintained below 2.5 Hz to preserve spectral resolution. Water suppression and spectral quality were optimized by adjusting the parameters for water resonance and 90° pulse length, thereby obtaining high‐resolution spectra. Spectral refinements were performed using Topspin software version 4.1 (Bruker, Ettlingen, Germany).

For spectral processing, Topspin was used to perform phasing, baseline correction, and calibration, aligning the TMSP resonance signal at δ0.0 ppm. Calibrated spectra were imported into Prometab (version 3.3) within MATLAB for spectral binning, region exclusion, peak alignment, and normalization (Viant, 2003). Spectral regions from δ0.2 to δ10 ppm were segmented into δ0.005‐ppm bins, excluding residual water and urea regions (δ4.1–δ6.2 ppm). Normalization to urinary creatinine was not applied because HS‐related dehydration or fluid imbalance may alter urine dilution and confound spot urinary creatinine concentrations. Therefore, normalization was performed by the total spectral area, a well‐established and robust normalization method in the field of metabolomics (Li et al., 2022; Wu & Li, 2016). The binned data were log‐transformed to reduce skewness and Pareto‐scaled to attenuate the dominance of highly variable metabolites while maintaining their relative importance; then, the processed data were subjected to multivariate chemometric analysis (van den Berg et al., 2006). Two NMR spectra were excluded because of the detection of abnormal metabolites in both pre‐ and postshift urine samples of the same participant. Urine samples were analyzed over multiple days. NMR spectra of the quality control samples were collected at the start of each day and the end of the final day.

### 2.4 Statistical Analysis

Pretreated binned spectral data were imported into SIMCA (version 13; Umetrics, San Jose, CA, USA) for multivariate analyses, such as principal component analysis and partial least squares discriminant analysis (PLS‐DA).

Principal component analysis is an unsupervised method that reduces the dimensionality of data sets; preserves the variability of data; and improves the visualization of patterns, trends, and group separation. Conversely, PLS‐DA is a supervised method that predicts class membership and enhances group separation by minimizing irrelevant variations. Model performance was evaluated in terms of the R^2^Y (explained variation) and Q^2^ (predictive accuracy) values. A higher R^2^Y value indicates a stronger model fit, and a negative Q^2^ value indicates overfitting or invalidity. Prior to statistical testing, the normality of the log‐transformed data for each metabolite was assessed using the Shapiro‐Wilk test. Wilcoxon signed rank and Mann‐Whitney U tests were performed to assess differences in the HCWs' urinary metabolite levels, with *P* values further adjusted via the Benjamini–Hochberg (BH) procedure to control the false discovery rate (Benjamini & Hochberg, 1995). Metabolites with *P* < 0.05 but BH‐adjusted *P* ≥ 0.05 were considered suggestive trends and retained for discussion to explore their potential roles in HS‐related IKI. Spearman's rank correlation test was used to assess associations among postshift urinary metabolites, blood creatinine, and blood pressure in the IKI population.

### 2.5 Metabolite Identification

Metabolites were identified by comparing chemical shifts, peak areas, and signal multiplicities in the ^1^H NMR spectra with reference data from a previous study (Piras et al., 2020), the Chenomx NMR Suite (Professional Edition; version 6.1; Chenomx, Canada), and the Human Metabolome Database (http://www.hmdb.ca).

## 3 Results

After work, nine HCWs were diagnosed as having IKI on the basis of elevated blood creatinine levels, consistent with the Kidney Disease: Improving Global Outcomes guidelines (Kidney Disease: Improving Global Outcomes Acute Kidney Injury Work Group, 2012). The median pre‐ and postshift creatinine levels were 0.7 and 1.2 mg/dL, respectively (Table 1). No significant differences were noted in sex, age, height, weight, body mass index, smoking status, electrolyte use in drinking water, sugary beverage intake, shift duration, duration in PPE, fluid intake, eGFR, or kinetic glomerular filtration rate, dehydration rate, preshift body temperature, and blood pressure between the IKI and non‐IKI populations (Table 1). Preshift blood creatinine levels did not differ significantly between the IKI and non‐IKI populations. However, median postshift body temperature was also significantly higher in the IKI population (36.8 vs. 36.5°C; *P* = 0.027). In addition, median postshift blood creatinine levels were significantly higher in the IKI population than in the non‐IKI population (1.2 vs. 0.9 mg/dL; *P* < 0.001).

**Table 1:** Demographic Characteristics of the IKI and Non‐IKI Populations

Variables	IKI population (*n* = 9)	Non‐IKI population (*n* = 29)	*P* Value
Women	9 (100)	25 (86)	0.248^a^
Age, years	29 (27–30)	28 (27–32)	0.425^b^
Height, cm	157 (155–158)	160 (158–165)	0.082^b^
Weight, kg	58.1 (48.5–61.9)	59.9 (52.0–65.9)	0.744^b^
Body mass index, kg/m^2^	23.5 (19.4–25.8)	21.6 (20.6–25.7)	0.959^b^
Current smoker	1 (11)	3 (10)	0.520^a^
Electrolytes used	1 (11.1)	12 (41.4)	0.095^a^
Sugary beverage	3 (33.3)	11 (37.9)	0.803^a^
Work shift, min	122.9 (106.4–128.2)	117.2 (111.8–126.7)	0.923^b^
Duration in PPE, min	120 (120–270)	120 (120–292.5)	0.844^b^
Fluid intake, mL	500 (300–600)	400 (0–600)	0.542^b^
kGFR, mL/min/1.73 m^2^	18.1 (16.5–19.3)	18.2 (16.8–20.8)	0.345^b^
eGFR, mL/min/1.73 m^2^	105.4 (100.5–119.2)	107.7 (93.8–116.5)	0.571^b^
Dehydration rate, n (%)^c^			
Before work	4 (44.4)	9 (31.0)	0.229^a^
After work	4 (44.4)	7 (24.1)	0.241^a^
Body temperature, °C			
Before work	36.4 (36.3–36.7)	36.3 (36.1–36.5)	0.276^b^
After work	36.8 (36.6–36.9)	36.5 (36.4–36.8)	0.027^b^
Blood creatinine level, mg/dL			
Before work	0.7 (0.7–0.8)	0.8 (0.7–0.9)	0.149^b^
After work	1.2 (1.1–1.3)	0.9 (0.8–1.0)	<0.001^b^
Systolic blood pressure, mmHg			
Before work	114 (107–118)	114 (104–121)	0.864^b^
After work	113 (108–115)	110 (101–115)	0.353^b^
Diastolic blood pressure, mmHg			
Before work	73 (73–79)	75 (70–81)	0.959^b^
After work	76 (70–83)	73 (67–77)	0.229^b^

*Note.* IKI, incident kidney injury; PPE, personal protective equipment; kGFR, kinetic glomerular filtration rate; eGFR, estimated glomerular filtration rate.

^a^ Chi‐square test.

^b^ Mann‐Whitney U test Data are presented in terms of number (%) and median (25–75 percentiles) values.

^c^ Dehydration rate: urine specific gravity ≥1.018 in the urine sample.

**Figure 1:**
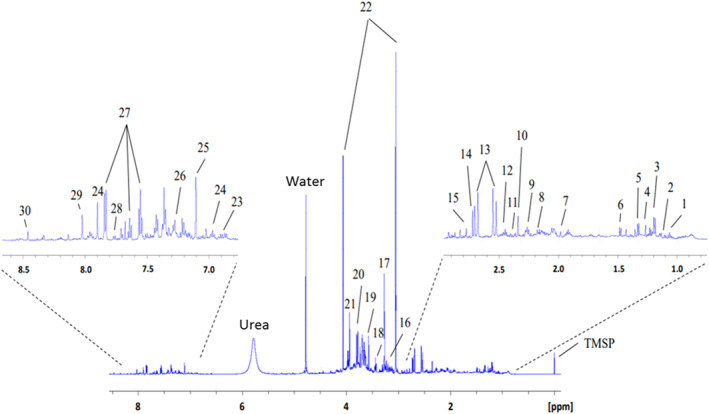
Urinary metabolic profiles of health‐care workers. Urine metabolic profiles were obtained through 600 MHz ^1^H nuclear magnetic resonance spectroscopy.

Figure 1 presents a representative 600 MHz ^1^H NMR spectrum depicting the urinary metabolic profiles of the study cohort. The experiment identified diverse urinary metabolites: 13 energy‐related metabolites, namely, 2‐oxoglutarate, 3‐hydroxyisovalerate, acetate, acetoacetate, alanine, citrate, creatine, formate, lactate, methylguanidine, methylmalonate, succinate, and valine; 9 oxidative stress–related metabolites, namely, 4‐hydroxybenzoate, adenine, glutarate, glycine, hippurate, kynurenine, taurine, trimethylamine N‐oxide (TMAO), and tryptophan; and 8 other metabolites, namely, acetone, creatinine, dimethylamine, guanidoacetate, histamine, histidine, isobutyrate, and phenylacetate. Table S1 in Supporting Information S1 presents the chemical shifts for these metabolites.

1. Valine, 2. Isobutyrate, 3. Methylmalonate, 4. 3‐Hydroxyisovalerate, 5. Lactate, 6. Alanine, 7. Acetate, 8. Acetone, 9. Glutarate, 10. Acetoacetate, 11. Succinate, 12. 2‐Oxoglutarate, 13. Citrate, 14. Dimethylamine, 15. Methylguanidine, 16. Taurine, 17. Trimethylamine N‐oxide, 18. Glycine, 19. Phenylacetate, 20. Guanidoacetate, 21. Creatine, 22. Creatinine, 23. 4‐Hydroxybenzoate, 24. Histamine, 25. Histidine, 26. Tryptophan, 27. Hippurate, 28. Kynurenine, 29. Adenine, 30. Formate.

A PLS‐DA score plot (Figure S1 in Supporting Information S1) showed a separation between pre‐ and postshift urine samples. Preshift samples (circles) and postshift samples (triangles) were mainly distributed in the negative and positive regions, respectively, along latent variable 1 (R^2^Y = 0.33; *Q* ^2^ = 0.06). Table 2 presents the pre‐ and postshift urinary metabolite levels of the study cohort. Significant postshift increases were observed in the levels of creatine (*P* = 0.006, BH‐adjusted *P* = 0.025), methylguanidine (*P* = 0.002, BH‐adjusted *P* = 0.018), and TMAO (*P* = 0.004, BH‐adjusted *P* = 0.025). By contrast, significant postshift reductions were noted in the levels of formate (*P* = 0.01, BH‐adjusted *P* = 0.033), methylmalonate (*P* = 0.001, BH‐adjusted *P* = 0.013), 4‐hydroxybenzoate (*P* = 0.005, BH‐adjusted *P* = 0.025), adenine (*P* < 0.001, BH‐adjusted *P* = 0.006), hippurate (*P* < 0.001, BH‐adjusted *P* = 0.001), and tryptophan (*P* = 0.008, BH‐adjusted *P* = 0.030). Additionally, postshift urinary valine levels among the HCWs trended higher (*P* = 0.043, BH‐adjusted *P* ≥ 0.05).

**Table 2:** Postshift Changes in the Urinary Metabolite Levels of Health‐Care Workers (*N* = 38)

Metabolites	Fold change^a^	Wilcoxon signed rank test (*P* value^b^)	BH‐adjusted *P* value^b^
Energy‐related metabolites			
2‐Oxoglutarate	0.992	0.694	0.890
3‐Hydroxyisovalerate	0.934	0.087	0.217
Acetate	0.976	0.423	0.746
Acetoacetate	0.952	0.925	0.962
Alanine	1.043	0.741	0.890
Citrate	0.969	0.561	0.801
Creatine	1.094	0.006**	0.025*
Formate	0.876	0.010**	0.033*
Lactate	1.030	0.875	0.962
Methylguanidine	1.216	0.002**	0.018*
Methylmalonate	0.892	0.001**	0.013*
Succinate	0.996	0.925	0.962
Valine	1.047	0.043*	0.128
Oxidative stress–related metabolites			
4‐Hydroxybenzoate	0.622	0.005**	0.025*
Adenine	0.619	<0.001***	0.006**
Glutarate	0.987	0.626	0.854
Glycine	1.015	0.423	0.746
Hippurate	0.652	<0.001***	0.001**
Kynurenine	0.793	0.192	0.444
Taurine	1.295	0.059	0.162
Trimethylamine N‐oxide	1.221	0.004**	0.025*
Tryptophan	0.863	0.008**	0.030*
Other metabolites			
Acetone	0.973	0.730	0.890
Creatinine	0.938	0.226	0.485
Dimethylamine	1.029	0.962	0.962
Guanidoacetate	0.948	0.470	0.783
Histamine	0.713	0.950	0.962
Histidine	1.029	0.499	0.788
Isobutyrate	1.029	0.251	0.503
Phenylacetate	1.067	0.551	0.801

*Note.* BH, Benjamini‐Hochberg.

^a^ Fold change: median of postshift/preshift levels.

^b^ Significance: *P < 0.05, **P < 0.01, and ***P < 0.001.

**Figure 2:**
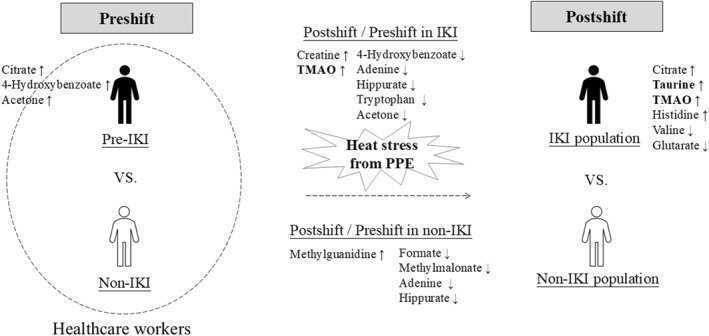
Heat stress–related changes in the urinary metabolite levels of health‐care workers with or without incident kidney injury. [↑] indicates increased fold change; [↓] indicates decreased fold change. Abbreviations: TMAO, Trimethylamine N‐oxide; PPE, personal protective equipment.

Preshift urinary metabolic profiles were compared between the IKI and non‐IKI populations (Figure 2; Table 3). Before work, the IKI population had significantly higher urinary citrate (*P* < 0.001, BH‐adjusted *P* = 0.008), and showed an observed trend (*P* < 0.05, but BH‐adjusted *P* ≥ 0.05) toward higher 4‐hydroxybenzoate (*P* = 0.009), and acetone (*P* = 0.01) levels compared to the non‐IKI population (Figure 2; Table 3). After work, the IKI population showed an observed trend (*P* < 0.05, but BH‐adjusted *P* ≥ 0.05) toward lower glutarate (*P* = 0.012) and valine (*P* = 0.034), and higher citrate (*P* = 0.006), taurine (*P* = 0.011), TMAO (*P* = 0.028), and histidine (*P* = 0.021) levels compared to the non‐IKI population (Figure 2; Table 3).

**Table 3:** Pre‐ and Postshift Levels of Urinary Metabolites in the IKI Population (*n* = 9) and Non‐IKI Population (*n* = 29)

Metabolites	Before work			After work		
	Fold change^a^	Mann‐whitney U test (*P* value^b^)	BH‐adjusted *P* value^b^	Fold change^a^	Mann‐whitney U test (*P* value^b^)	BH‐adjusted *P* value^b^
Energy‐related metabolites						
2‐Oxoglutarate	1.019	0.784	0.937	0.889	0.119	0.325
3‐Hydroxyisovalerate	0.994	0.812	0.937	0.928	0.339	0.442
Acetate	1.010	0.499	0.937	0.944	0.339	0.442
Acetoacetate	0.951	0.547	0.937	0.952	0.103	0.310
Alanine	0.996	0.784	0.937	0.973	0.479	0.575
Citrate	1.055	<0.001***	0.008**	1.214	0.006**	0.120
Creatine	0.970	0.622	0.937	1.160	0.096	0.310
Formate	1.001	0.523	0.937	1.040	0.272	0.430
Lactate	0.941	0.149	0.684	0.930	0.147	0.338
Methylguanidine	0.908	0.265	0.937	0.837	0.052	0.221
Methylmalonate	0.930	0.298	0.937	0.856	0.083	0.310
Succinate	1.003	0.701	0.937	0.929	0.167	0.359
Valine	0.966	0.160	0.684	0.861	0.034*	0.168
Oxidative stress–related metabolites						
4‐Hydroxybenzoate	1.466	0.009**	0.100	1.021	0.697	0.697
Adenine	0.992	0.499	0.937	0.868	0.272	0.430
Glutarate	0.954	0.160	0.684	0.795	0.012*	0.120
Glycine	1.011	0.391	0.937	0.898	0.547	0.586
Hippurate	0.942	0.985	0.985	0.910	0.571	0.591
Kynurenine	1.053	0.729	0.937	0.958	0.257	0.430
Taurine	0.972	0.927	0.985	1.315	0.011*	0.120
Trimethylamine N‐oxide	1.031	0.729	0.937	1.255	0.028*	0.168
Tryptophan	1.021	0.391	0.937	0.924	0.339	0.442
Other metabolites						
Acetone	1.150	0.010*	0.100	1.022	0.501	0.578
Creatinine	1.000	0.523	0.937	0.950	0.229	0.429
Dimethylamine	0.999	0.729	0.937	1.030	0.547	0.586
Guanidoacetate	1.013	0.985	0.985	0.900	0.147	0.338
Histamine	0.816	0.985	0.985	2.621	0.288	0.432
Histidine	1.211	0.139	0.684	1.309	0.021*	0.160
Isobutyrate	1.007	0.648	0.937	0.925	0.416	0.519
Phenylacetate	0.997	0.756	0.937	1.149	0.229	0.429

*Note.* IKI, incident kidney injury; BH, Benjamini‐Hochberg.

^a^ Fold change: median of IKI/non‐IKI populations.

^b^ Significance: *P < 0.05 and **P < 0.01.

Further analysis of the IKI and non‐IKI populations showed differences in metabolic profiles. PLS‐DA of pre‐ and postshift urine samples in the IKI population showed a separation along latent variable 1 (R^2^Y = 0.80; *Q* ^2^ = 0.05; Figure S2 in Supporting Information S1). Significant within‐group differences were observed in urinary metabolic profiles before and after work (Figure 2; Table 4). In the IKI population, postshift urine samples exhibited a trend (*P* < 0.05, but BH‐adjusted *P* ≥ 0.05) toward higher levels of creatine (*P* = 0.011) and TMAO (*P* = 0.011) but lower levels of 4‐hydroxybenzoate (*P* = 0.008), adenine (*P* = 0.015), hippurate (*P* = 0.021), tryptophan (*P* = 0.008), and acetone (*P* = 0.021), compared to preshift samples (Table 4). In the non‐IKI population, postshift urine samples exhibited significantly lower hippurate levels (*P* < 0.001, BH‐adjusted *P* = 0.018). Additionally, observed trends (*P* < 0.05, but BH‐adjusted *P* ≥ 0.05) were found toward lower levels of formate (*P* = 0.032), methylmalonate (*P* = 0.006), and adenine (*P* = 0.013), but a higher level of methylguanidine (*P* = 0.009), compared to preshift samples (Table 4).

**Table 4:** Heat Stress–Related Changes in Urinary Metabolite Levels of the IKI Population (*n* = 9) and Non‐IKI Population (*n* = 29)

Metabolites	IKI population			Non‐IKI population		
	Fold change^a^	Wilcoxon signed rank test (*P* value^b^)	BH‐adjusted *P* value^b^	Fold change^a^	Wilcoxon signed rank test (*P* value^b^)	BH‐adjusted *P* value^b^
Energy‐related metabolites						
2‐Oxoglutarate	0.885	0.515	0.735	1.006	0.923	0.981
3‐Hydroxyisovalerate	0.899	0.594	0.810	0.938	0.090	0.423
Acetate	0.910	0.515	0.735	1.006	0.113	0.423
Acetoacetate	1.007	0.374	0.591	0.924	0.728	0.961
Alanine	1.029	0.953	0.953	1.050	0.737	0.961
Citrate	0.872	0.374	0.591	0.998	0.981	0.981
Creatine	1.276	0.011*	0.081	1.031	0.153	0.459
Formate	0.888	0.173	0.458	0.838	0.032*	0.184
Lactate	1.034	0.859	0.920	0.995	0.981	0.981
Methylguanidine	1.222	0.214	0.458	1.155	0.009**	0.062
Methylmalonate	0.845	0.110	0.329	0.846	0.006**	0.062
Succinate	0.931	0.214	0.458	1.007	0.424	0.707
Valine	1.089	0.260	0.521	1.098	0.124	0.423
Oxidative stress–related metabolites						
4‐Hydroxybenzoate	0.419	0.008***	0.081	0.899	0.542	0.654
Adenine	0.546	0.015*	0.089	0.651	0.013*	0.066
Glutarate	0.936	0.767	0.852	1.058	0.456	0.752
Glycine	0.822	0.767	0.852	1.051	0.212	0.488
Hippurate	0.675	0.021*	0.089	0.626	<0.001***	0.018*
Kynurenine	0.689	0.066	0.221	0.858	0.790	0.981
Taurine	1.644	0.051	0.190	1.192	0.501	0.752
Trimethylamine N‐oxide	1.291	0.011*	0.081	1.091	0.130	0.434
Tryptophan	0.776	0.008**	0.081	0.910	0.374	0.492
Other metabolites						
Acetone	0.854	0.021*	0.089	1.109	0.186	0.488
Creatinine	0.907	0.678	0.848	0.941	0.212	0.488
Dimethylamine	1.054	0.767	0.852	1.020	0.876	0.981
Guanidoacetate	0.841	0.374	0.591	1.004	0.885	0.981
Histamine	1.965	0.374	0.591	0.481	0.799	0.806
Histidine	1.142	0.953	0.953	1.153	0.313	0.752
Isobutyrate	0.992	0.678	0.848	1.086	0.307	0.626
Phenylacetate	1.214	0.214	0.458	1.048	0.990	0.981

*Note.* IKI, incident kidney injury; BH, Benjamini‐Hochberg.

^a^ Fold change: median of postshift/preshift.

^b^ Significance: *P < 0.05, **P < 0.01, and ***P < 0.001.

## 4 Discussion

**Figure 3:**
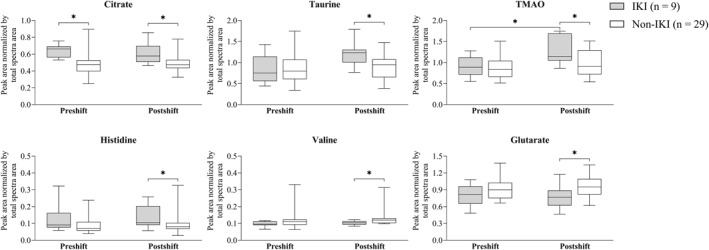
Postshift changes in six key urinary metabolites in health‐care workers with or without incident kidney injury. Abbreviations: TMAO, Trimethylamine N‐oxide. Significant *P* value: *P < 0.05, **P < 0.01. Box‐and‐whisker plots show the values of minimum, 25th percentile, median, 75th percentile, and maximum.

Understanding the potential molecular clues associated with IKI and its association with HS is crucial for identifying biomarkers and developing strategies for early detection and prevention of HS‐related kidney injury. During the COVID‐19 pandemic, HCWs had an increased risk of IKI, potentially related to experiencing prolonged HS from PPE use. To the best of our knowledge, this study is the first to investigate PPE use–related changes in the urinary metabolic profiles of HCWs. Postshift urinary taurine, TMAO, and histidine levels showed a trend (*P* < 0.05, but BH‐adjusted *P* ≥ 0.05) toward being higher in the IKI population than in the non‐IKI population; however, preshift levels of these metabolites did not differ significantly between the two populations (Figure 3). Furthermore, compared with the non‐IKI population, the IKI population exhibited elevated citrate levels both before and after work and lower glutarate and valine levels after work (Figure 3).

### 4.1 Associations of Taurine and TMAO With HS and Kidney Function

**Figure 4:**
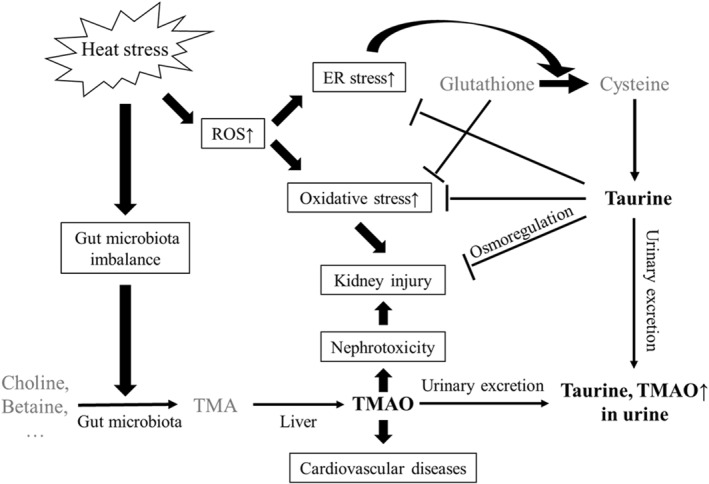
Potential roles of taurine and trimethylamine N‐oxide (TMAO) in the development of heat stress‐related kidney injury.

Taurine and TMAO are metabolites involved in cellular osmoregulation and antioxidant defense (Chesney et al., 2010; Zixin et al., 2022). HS has been associated with pathways that may be related to gut microbial changes, oxidative stress, and endoplasmic reticulum (ER) stress (Figure 4). Taurine is known to support kidney function, potentially by regulating blood pressure, alleviating oxidative stress, and mitigating inflammation (Baliou et al., 2020; Chen et al., 2023; Guan & Miao, 2020). HS is related to oxidative stress, which may contribute to kidney injury (Chapman et al., 2021). Our study revealed a significant postshift increase in the urinary taurine level of the IKI population, which may reflect taurine's role in scavenging reactive oxygen species (ROS) and stabilizing mitochondrial function (Jong et al., 2021).

Furthermore, a previous study showed that taurine is related to blood pressure regulation (Guan & Miao, 2020), which raised the possibility that our findings may merely reflect hemodynamic changes. Hence, we performed a correlation analysis. The results of our analysis showed no significant differences in preshift or postshift blood pressure between the IKI and non‐IKI populations (Table 1). Moreover, postshift urinary taurine levels in the IKI population showed no significant correlation with either postshift systolic or diastolic blood pressures (Table S2 in Supporting Information S1). In contrast, we observed a significant positive correlation between postshift urinary taurine and postshift blood creatinine levels (rs = 0.717, *P* = 0.030; Table S2 in Supporting Information S1) in the IKI population. Therefore, higher urinary taurine levels are more likely associated with kidney dysfunction rather than being solely a consequence of hemodynamic changes. However, the extent of taurine's protective capacity under prolonged HS remains unclear, warranting further research.

Taurine and TMAO may serve as potential indicators of HS‐related kidney injury. HS‐associated dysbiosis of the gut microbiota may facilitate the conversion of dietary compounds such as choline and betaine into trimethylamine (TMA), which is subsequently oxidized into TMAO in the liver. Elevated TMAO levels are associated with nephrotoxicity. HS has been related to increased production of ROS, potentially contributing to oxidative stress and ER stress, thereby exacerbating cellular damage and kidney injury. Glutathione is known to alleviate oxidative stress. Under oxidative stress conditions, cells may increasingly utilize cysteine, which is essential for the synthesis of both glutathione and taurine, potentially modulating taurine production. Taurine plays key roles in osmoregulation and cellular stress response. Thus, elevated urinary levels of taurine and TMAO may be associated with the development of HS‐related kidney injury.

TMAO, a metabolite produced by gut microbiota through the processing of dietary choline and carnitine, is associated with kidney dysfunction and cardiovascular diseases (Tang et al., 2023; Ufnal et al., 2015). This association may be attributable to intestinal barrier disruption and systemic inflammation, which are exacerbated by HS (Tang et al., 2023). Chicas et al. demonstrated that high HS alters gut microbiota–derived metabolites in agricultural workers; the researchers further suggested that exposure to pesticides, particularly glyphosate, can contribute to this alteration (Chicas et al., 2023). However, the cross‐sectional nature of their study precluded the establishment of causality and the adjustment of potential confounders, such as pesticide exposure. Our study observed a significant postshift increase in the urinary level of TMAO, suggesting a potential association between HS and alterations in gut microbiota; this finding is consistent with that of an animal study (Xia et al., 2022).

Based on previous findings, HS may be related to increased ROS levels and ER stress, potentially associated with oxidative stress and kidney injury (Tang et al., 2023) (Figure 4). NMR spectroscopy–based metabolomic analyses facilitate unbiased measurement of metabolites in human biofluids and the investigation of their correlations with phenotypes. However, few studies have used metabolomics to examine HS‐related kidney injury. A study involving heat‐stressed agricultural workers revealed increased plasma levels of citrulline and uracil, which were suggested to indicate kidney dysfunction (Chicas et al., 2023). Altered alanine and threonine levels in these workers were thought to indicate metabolic stress, whereas increased methylhistidine levels suggested oxidative stress (Chicas et al., 2023). The findings suggest that HS, in addition to other occupational factors, may be associated with alterations in workers' metabolic profiles (Chicas et al., 2023).

Taurine may alleviate oxidative stress (Chesney et al., 2010; Jong et al., 2021), whereas TMAO may exacerbate nephrotoxicity (Ufnal et al., 2015; Zixin et al., 2022). The increases in the urinary levels of taurine and TMAO during HS suggest a potential compensatory response involving taurine against oxidative damage, whereas TMAO may reflect an increased risk of kidney injury. Oxidative stress and energy metabolism have been implicated in the development of AKI (Martin‐Lorenzo et al., 2017). Further studies are needed to clarify the combined effects of taurine and TMAO on HS‐related kidney injury.

### 4.2 Citrate, Acetone, 4‐Hydroxybenzoate, and Glutarate Levels in the Study Cohort

Citrate plays an important role in energy production and metabolic regulation (Bender & Mayes, 2018). Increased urinary citrate levels can be attributable to citrate‐rich food intake, metabolic alkalosis, and impaired kidney function, which reduces citrate reabsorption (Hamm, 1990). In our study, both pre‐ and postshift urinary citrate levels were significantly higher in the IKI population than in the non‐IKI population. Notably, HCWs who developed IKI after HS had exhibited higher urinary citrate levels at baseline, but no other biochemical or physiological abnormalities. This finding is consistent with that of Tyson et al. (2021), who reported that patients with chronic kidney disease exhibited significant increases in urinary citrate levels after bicarbonate supplementation—a response not observed in individuals without chronic kidney disease (Tyson et al., 2021). Tyson et al. suggested that individuals with compromised kidney function may have impaired acid–base homeostasis, which increases citrate excretion in response to alkali (Tyson et al., 2021). We also observed that baseline levels of the ketone body acetone were higher in the pre‐IKI population than in the non‐IKI population. A previous study has indicated that ketogenesis can be enhanced under systemic alkalosis (Hood et al., 1990), potentially indicating the high alkaline state in the pre‐IKI population. Similarly, our findings suggest that citrate metabolism appears to be associated with kidney function. The mechanisms of a higher alkaline state are unclear. One possible explanation involves endocrine regulation where several hormones play a role in maintaining acid–base homeostasis (Wagner, 2014). Further research is needed to determine whether HCWs with IKI begin their shifts in a relatively high alkaline state.

Additionally, the pre‐IKI population exhibited a trend toward higher baseline levels of 4‐hydroxybenzoate in our study. 4‐Hydroxybenzoate is recognized as an endogenous product of tyrosine metabolism (Pierrel, 2017) and can also be derived from environmental sources (Moos et al., 2016). This finding, along with the higher baseline citrate and acetone levels, further supports the notion that a distinct pre‐existing metabolic profile, potentially related to a higher alkaline state, is associated with susceptibility to HS‐related kidney injury.

In our study, urinary glutarate levels significantly increased after HS in the non‐IKI population but not in the IKI population (Table 3; Figure 3), suggesting potential metabolic adaptations or protective mechanisms in the non‐IKI population. Glutarate, a metabolite derived from lysine and tryptophan catabolism (Borsook et al., 1948), is associated with renal protection in patients with hypertension. Accelerated lysine metabolism has been related to renal protection in patients with salt‐sensitive hypertension by enhancing the conjugation and excretion of lysine‐derived metabolites (Rinschen et al., 2022). The absence of significant postshift changes in the glutarate levels of HCWs with IKI may reflect a reduced compensatory response. Lysine metabolites, such as Nε‐malonyl‐lysine, may confer renal protection by depleting central carbon metabolites and modulating fatty acid metabolism (Rinschen et al., 2022), which is potentially related to metabolic stability in individuals without IKI. Further research is needed to identify factors that regulate glutarate levels under environmental stress conditions.

### 4.3 HS‐Related Metabolic Changes in the Study Cohort

Postshift changes were noted in the energy‐ and oxidative stress–related urinary metabolites of the study cohort. A strong model fit (R^2^Y = 0.80) but limited predictive capacity (*Q* ^2^ = 0.05) was noted in the IKI population (Figure S2 in Supporting Information S1), likely because of interpersonal variability and the complex metabolic responses to HS in the small sample size. However, the IKI population exhibited a trend in postshift changes in metabolites such as creatine, TMAO, 4‐hydroxybenzoate, adenine, hippurate, tryptophan, and acetone (Table 4), suggesting potential metabolic adaptations to HS.

Creatine is primarily synthesized in the kidneys and liver (Wyss & Kaddurah‐Daouk, 2000). In muscle tissue, creatine spontaneously cyclizes to form creatinine, which is excreted through urine (Wyss & Kaddurah‐Daouk, 2000). An in vitro study demonstrated that this reversible, nonenzymatic cycle favors creatine at elevated pH levels and low temperatures (Wyss & Kaddurah‐Daouk, 2000). Compared with our non‐IKI population, the IKI population had significantly higher urinary creatine levels after work, which may reflect a potential tendency toward alkalinity in acid–base homeostasis, potentially associated with HS. This contrasts with the non‐IKI population, in which creatine levels remained unchanged, but the downstream metabolite methylguanidine increased significantly. Notably, baseline urinary citrate levels were also higher in the IKI population than in the non‐IKI population; this finding suggests a potential preexisting alkaline tendency of patients with IKI, which may be correlated with HS susceptibility.

In the IKI population, postshift urinary levels of adenine and 4‐hydroxybenzoate were significantly lower than their preshift levels (Table 4). A study involving a rat model of diabetic nephropathy demonstrated that endogenous adenine enhanced extracellular matrix production in renal tubular cells; its inhibition mitigated kidney injury (Sharma et al., 2023). The benzoic acid–derivative 4‐hydroxybenzoate is essential for the biosynthesis of ubiquinone and adenosine triphosphate (Alam et al., 1975). In patients with AKI, urinary kynurenine levels are typically elevated because of reduced filtration and altered tryptophan metabolism (Zakrocka & Załuska, 2022). This finding is inconsistent with ours, likely because HS may be associated with altered metabolic pathways only in patients with IKI. Future studies should explore the correlations of these metabolites with HS‐related kidney injury. The changes in tryptophan and hippurate levels in response to heat exposure in the IKI population may further reflect gut microbiota dysregulation. Both metabolites can be related to microbial metabolic processes (Agus et al., 2018; Pallister et al., 2017). Won et al. noted decreased urinary hippurate and increased formate levels in AKI rats (Won et al., 2016). However, we found that postshift urinary levels of hippurate and formate were relatively low in the non‐IKI population (Table 4), potentially reflecting metabolic adaptations to HS, rather than kidney dysfunction. Similarly, postshift urinary levels of methylmalonate were lower in the non‐IKI population than in the IKI population (Table 4). However, the decrease in formate contrasts with the increase reported in AKI rat models (Won et al., 2016), suggesting these changes may be part of an adaptive metabolic response to HS rather than a sign of kidney dysfunction. Methylmalonate is a primary biomarker for vitamin B12 deficiency; blood levels of methylmalonate are influenced by vitamin B12 status, eGFR, age, sex, metabolic rate, diet, and gut microbiota (Riphagen et al., 2020). Individuals with impaired kidney function often have elevated serum methylmalonate levels (Riphagen et al., 2020). Our study revealed potential associations between HS‐related metabolic changes and kidney injury.

## 5 Limitations

This study has several limitations. First, postshift metabolic changes may be associated with factors beyond HS. The potential biomarkers of HS‐related IKI in HCWs should be further evaluated through animal studies. Second, this was an exploratory, hypothesis‐generating investigation with a modest sample size; the size of the IKI population was particularly low. Third, considering the need for a minimally invasive protocol for our frontline HCW cohort, the measurements were performed using urine samples. However, other biological materials, such as blood and feces, may provide comprehensive information. Additional clinical data, such as imaging results and kidney biopsy findings, may help determine the type and location of kidney injury. Similarly, a detailed dietary assessment and a direct measurement of work intensity were not performed, as we assumed those HCWs were performing similar tasks. However, these are important variables for consideration in future studies. The study is underpowered to detect modest but meaningful differences between the IKI and non‐IKI populations. Furthermore, the limited number of IKI events restricted multivariable regression adjustment for potential confounders in the metabolite analyses. Additionally, because we examined numerous metabolites, most findings showed an observed trend (*P* < 0.05, but BH‐adjusted *P* ≥ 0.05) rather than meeting BH‐adjusted significance. Therefore, the possibility of residual confounding from unmeasured factors remains a concern. Results for IKI versus non‐IKI populations are exploratory and descriptive rather than inferential or confirmatory. Future large‐scale studies with prespecified adjustment strategies are needed to validate these findings. Finally, some metabolites identified in our study are correlated with gut microbiota–derived metabolites (Chicas et al., 2023; Riphagen et al., 2020; Tang et al., 2023; Ufnal et al., 2015). Thus, future studies should integrate microbiota and metabolomic analyses.

## 6 Conclusions

We investigated PPE use–related urinary metabolic abnormalities in HCWs, identifying potential urinary metabolic pertubations associated with HS‐related IKI. The increases in the urinary TMAO levels during HS provide descriptive signals suggesting that TMAO may be associated with an increased risk of kidney injury. Additionally, postshift urinary levels of taurine showed a trend toward being higher in the IKI population, which suggests that taurine may reflect a physiological response to oxidative damage. Thus, further exploring oxidative stress may inform future strategies for preventing HS‐related kidney injury. We further noted that baseline urinary citrate, acetone, and 4‐hydroxybenzoate levels were relatively high in the IKI population, suggesting a possible preexisting alkaline tendency and potential susceptibility to environmental exposures. However, postshift glutarate levels were higher in the non‐IKI population than in the IKI population, potentially reflecting adaptive or protective mechanisms in the non‐IKI population. Because these findings are hypothesis‐generating, future large‐scale or animal studies are needed to verify these observations and establish causality. Improving working conditions for HCWs in hot environments, such as optimizing PPE for improved heat dissipation, may reduce the risk of HS‐related kidney injury.

## Conflict of Interest

The authors declare no conflicts of interest relevant to this study.

## Supporting information

Supporting Information S1

## Data Availability

The data sets generated and analyzed during this study are not publicly available due to privacy and ethical restrictions concerning human subject data. Data may be made available from the corresponding author upon reasonable request. The software used for data analysis included Topspin version 4.1 (Bruker), Prometab version 3.3 within MATLAB, and SIMCA version 13 (Umetrics).
