# Supplementary material for: Nuclear Magnetic Resonance–Based Urinary Metabolic Profiling for Assessing Heat Stress–Related Incident Kidney Injury in Health‐Care Workers
Source: Geohealth. 2026 Jun 2;10(6):e2025GH001583. doi: 10.1029/2025GH001583 (PMC13238615; doi:10.1029/2025GH001583)
Supplement: Supplementary file 1 — Supporting Information S1 [file GH2-10-e2025GH001583-s001.docx]

*GeoHealth*

Supporting Information for

**Nuclear Magnetic Resonance–Based Urinary Metabolic Profiling for Assessing Heat Stress–Related Incident Kidney Injury in Health-Care Workers**

Cheng-Yu Ting^1,#^, Chi-Tsung Chen^1,2,#^, Tzu-Hsin Yen^1^, Jason Kai Wei Lee^3,4,5^, Sheng-Han Lee^6^, Shang-Jen Chang^7^, Tzu-Han Hung^1^, Hao-Jan Liang^1^, Hsiao-Yu Yang^1,8,*^, and Ching-Yu Lin^1,8,*^

^1^Institute of Environmental and Occupational Health Sciences, College of Public Health, National Taiwan University, Taipei, Taiwan.

^2^Department of Respiratory Care, Shin Kong Wu Ho Su Memorial Hospital, Taipei City, Taiwan.

^3^Heat Resilience and Performance Centre, Yong Loo Lin School of Medicine, National University of Singapore, Singapore.

^4^Human Potential Translational Research Programme, Yong Loo Lin School of Medicine, National University of Singapore, Singapore.

^5^Department of Physiology, Yong Loo Lin School of Medicine, National University of Singapore, Singapore.

^6^School of Medicine, College of Medicine, National Sun Yat-Sen University, Kaohsiung, Taiwan.

^7^Department of Urology, National Taiwan University Hospital, Taipei, Taiwan.

^8^Population Health Research Center, National Taiwan University, Taipei, Taiwan.

^#^These authors contributed equally to this work.

^*^Corresponding author: Hsiao-Yu Yang (hyang@ntu.edu.tw), Ching-Yu Lin (chingyulin@ntu.edu.tw)

**Contents of this file**

Tables S1 to S2

Figures S1 to S2

**Introduction**

This document aims to complement key data-processing results and justify the robustness of the methods used in the main manuscript.

Table S1. Metabolites identified in the urine of the health-care workers by 600 MHz ^1^H nuclear magnetic resonance.

| Metabolites | Chemical shift (multiplicity^a^) |
| --- | --- |
| Valine | 1.04(d)^#^, 2.26(m), 3.61(d) |
| Isobutyrate | 1.07(d) |
| Methylmalonate | 1.23(d) |
| 3-Hydroxyisovalerate | 1.27(s)^#^, 2.34(s) |
| Lactate | 1.33(d) |
| Alanine | 1.47(d)^#^, 3.82(q) |
| Acetate | 1.98(s) |
| Acetone | 2.10(s) |
| Glutarate | 2.27(t) |
| Acetoacetate | 2.29(s)^#^, 3.46(s) |
| Succinate | 2.42(s) |
| 2-Oxoglutarate | 2.46(t) |
| Citrate | 2.56(d)^#^, 2.69(d) |
| Dimethylamine | 2.73(s) |
| Methylguanidine | 2.86(s)^#^, 3.36(s) |
| Creatinine | 3.02(s), 4.05(s)^#^ |
| Creatine | 3.06(s), 3.96(s)^#^ |
| Trimethylamine N-oxide | 3.27(s) |
| Taurine | 3.27(t), 3.43(t)^#^ |
| Glycine | 3.52(s) |
| Phenylacetate | 3.56(s)^#^, 7.32(m), 7.37(m) |
| Guanidoacetate | 3.78(s) |
| Hippurate | 4.01(d), 7.63(t)^#^, 7.91(d) |
| 4-Hydroxybenzoate | 6.97(d) |
| Histamine | 7.08(s) |
| Histidine | 7.16(s)^#^, 7.98(s) |
| Tryptophan | 7.32(s)^#^, 7.55(d), 7.78(d) |
| Kynurenine | 7.76(d) |
| Adenine | 8.10(s) |
| Formate | 8.46(s) |
| ^a^: Singlet (s), doublet (d), triplet (t), quartet (q), mutiplet (m)  ^#^: Chemical shift used for quantification. | |

**Table S2.** Spearman's correlation matrix of postshift urinary metabolites, blood pressure, and blood creatinine in the incident kidney injury population (n=9).

| Variables | (1) | (2) | (3) | (4) | (5) | (6) | (7) | (8) | (9) |
| --- | --- | --- | --- | --- | --- | --- | --- | --- | --- |
| 1. Systolic blood pressure | 1 |  |  |  |  |  |  |  |  |
| 1. Diastolic blood pressure | 0.823** | 1 |  |  |  |  |  |  |  |
| 1. Blood creatinine | 0.378 | 0.385 | 1 |  |  |  |  |  |  |
| Urinary metabolites | | | | | | | | | |
| 1. Citrate | -0.622 | -0.552 | 0.100 | 1 |  |  |  |  |  |
| 1. Taurine | 0.429 | 0.259 | 0.717* | 0.017 | 1 |  |  |  |  |
| 1. Trimethylamine N-oxide | 0.210 | 0.033 | -0.417 | -0.517 | 0.200 | 1 |  |  |  |
| 1. Histidine | 0.118 | 0.469 | -0.100 | -0.150 | -0.233 | -0.183 | 1 |  |  |
| 1. Valine | -0.364 | -0.342 | -0.227 | -0.143 | -0.513 | -0.202 | -0.395 | 1 |  |
| 1. Glutarate | 0.076 | 0.218 | 0.000 | -0.050 | -0.217 | -0.283 | -0.183 | 0.420 | 1 |
| ** P* < 0.05, ** *P* < 0.01 | | | | | | | | | |


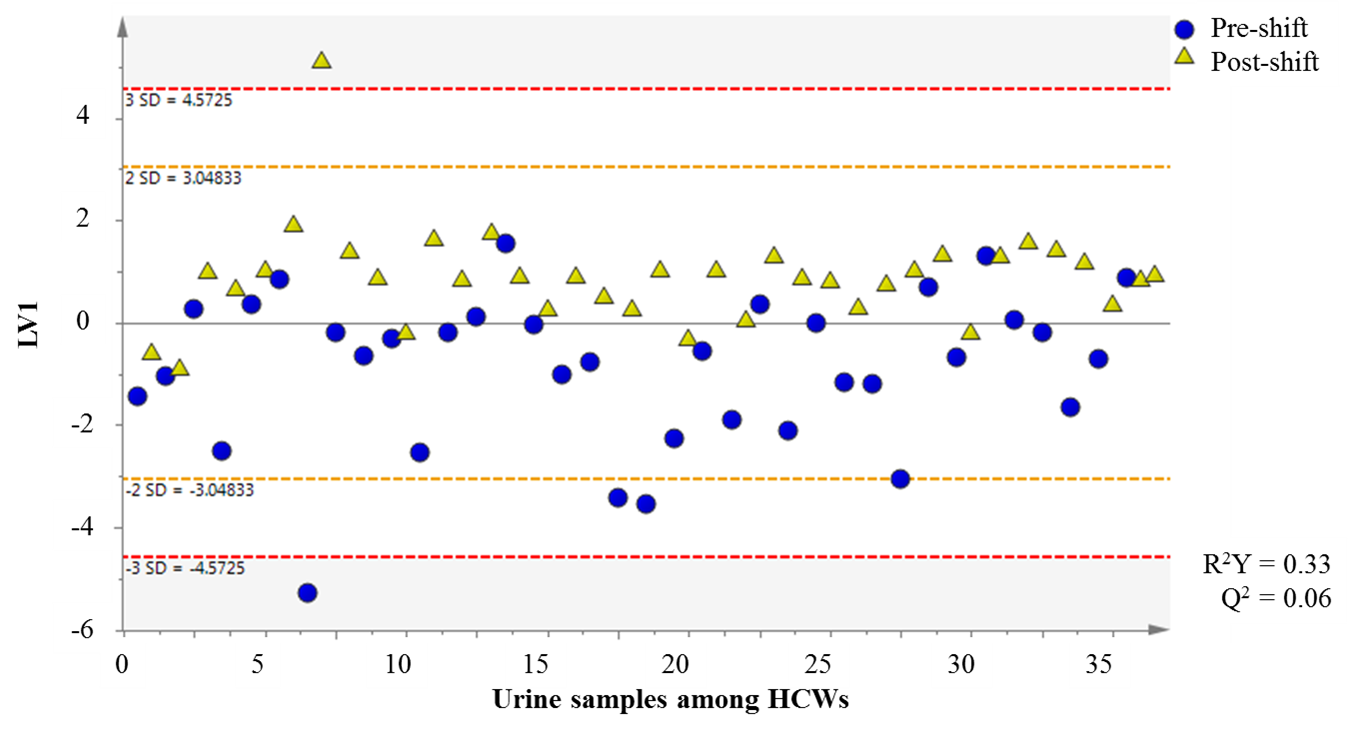


**Figure S1.** Partial least squares discriminant analysis scores plot from the analysis of the ^1^H nuclear magnetic resonance spectra of pre- and postshift urine samples among health-care workers (HCWs).


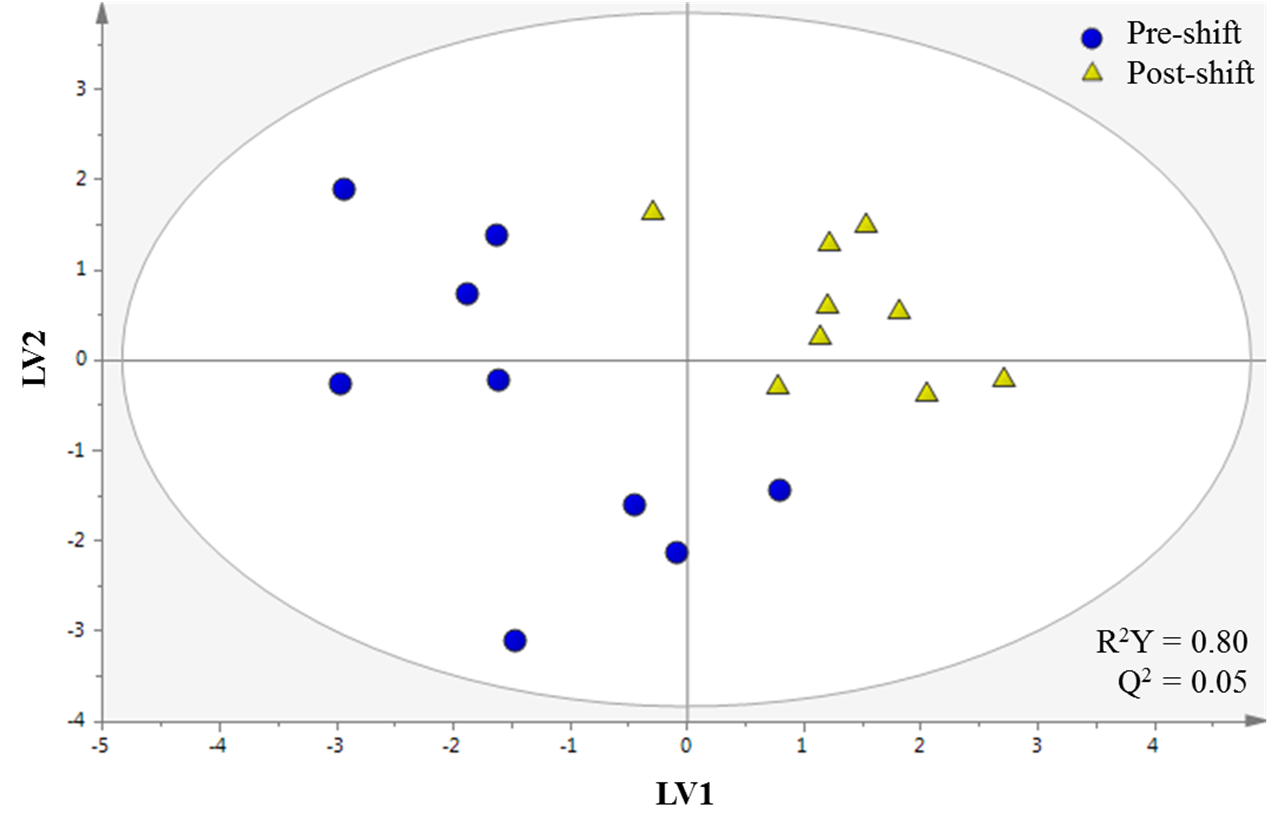


**Figure S2.** Partial least squares discriminant analysis scores plot from the analysis of ^1^H nuclear magnetic resonance spectra in the population with incident kidney injury.
